# SUPPORT Tools for evidence-informed health Policymaking (STP) 15: Engaging the public in evidence-informed policymaking

**DOI:** 10.1186/1478-4505-7-S1-S15

**Published:** 2009-12-16

**Authors:** Andrew D Oxman, Simon Lewin, John N Lavis, Atle Fretheim

**Affiliations:** 1 Norwegian Knowledge Centre for the Health Services, P.O. Box 7004, St. Olavs plass, N-0130 Oslo, Norway; 2 Norwegian Knowledge Centre for the Health Services, P.O. Box 7004, St. Olavs plass, N-0130 Oslo, Norway; Health Systems Research Unit, Medical Research Council of South Africa; 3 Centre for Health Economics and Policy Analysis, Department of Clinical Epidemiology and Biostatistics, and Department of Political Science, McMaster University, 1200 Main St. West, HSC-2D3, Hamilton, ON, Canada, L8N 3Z5; 4 Norwegian Knowledge Centre for the Health Services, P.O. Box 7004, St. Olavs plass, N-0130 Oslo, Norway; Section for International Health, Institute of General Practice and Community Medicine, Faculty of Medicine, University of Oslo, Norway

## Abstract

*This article is part of a series written for people responsible for making decisions about health policies and programmes and for those who support these decision makers*.

In this article, we address strategies to inform and engage the public in policy development and implementation. The importance of engaging the public (both patients and citizens) at all levels of health systems is widely recognised. They are the ultimate recipients of the desirable and undesirable impacts of public policies, and many governments and organisations have acknowledged the value of engaging them in evidence-informed policy development. The potential benefits of doing this include the establishment of policies that include their ideas and address their concerns, the improved implementation of policies, improved health services, and better health. Public engagement can also be viewed as a goal in itself by encouraging participative democracy, public accountability and transparency. We suggest three questions that can be considered with regard to public participation strategies. These are: 1. What strategies can be used when working with the mass media to inform the public about policy development and implementation? 2. What strategies can be used when working with civil society groups to inform and engage them in policy development and implementation? 3. What methods can be used to involve consumers in policy development and implementation?

## About STP

*This article is part of a series written for people responsible for making decisions about health policies and programmes and for those who support these decision makers. The series is intended to help such people ensure that their decisions are well-informed by the best available research evidence. The SUPPORT tools and the ways in which they can be used are described in more detail in the Introduction to this series *[1]. *A glossary for the entire series is attached to each article (see Additional File *1*). Links to Spanish, Portuguese, French and Chinese translations of this series can be found on the SUPPORT website *http://www.support-collaboration.org. *Feedback about how to improve the tools in this series is welcome and should be sent to*: STP@nokc.no.

## Scenario

*The Minister of Health has promised to deliver a new healthcare reform. In declaring her intentions, the Minister has emphasised the importance of engaging stakeholders in the development of the proposal for the reform. You are a member of the team responsible for developing the proposal and for ensuring that key stakeholders are informed about relevant research evidence and engaged effectively in evidence-informed policy development*.

## Background

In this article, we present three questions that policymakers and those who support them can ask when considering strategies to inform and engage the public in evidence-informed policy development and implementation, such as in the scenario described above.

Much of the terminology used to describe individuals who come into contact with health systems is problematic [2,3]. Words such as 'patient', 'client', 'consumer' and 'user' are commonly used, but may be misleading or considered unacceptable by those they are applied to. Several of these terms, for example, implicitly suggest the existence of a market-based relationship and some people may find this objectionable. Nonetheless, the term 'consumer' is commonly used when describing approaches that engage people in decisions about healthcare [4,5].

Healthcare 'consumers' can include patients, unpaid carers, parents or guardians of patients, users of health services, disabled people, members of the public who are the potential recipients of either health promotion or public health programmes, people who believe they have been exposed to potentially harmful products or services, people who believe they have been denied products or services which they believe could have benefited them, as well as those who pay for health services (e.g. as tax payers) [6]. Depending on the context, people can be described as 'lay' people, 'non-experts', 'service users', 'members of the general public' or as 'citizens'. In this article, we use the term 'the public' to include people in any of these various roles, and the term 'consumer' when referring to *individuals *in any of these roles.

The importance of engaging the public at all levels of health systems is widely recognised. This is because members of the public are the ultimate recipients of the effects of health policy, both intended and unintended, and many governments and organisations have acknowledged the value of public engagement in policy development. The potential benefits of doing this include the development of policies that include their ideas or address their concerns, the improvement of policy implementation, better health services, and better health outcomes.

Public engagement can also be viewed as a goal in itself by encouraging participative democracy, public accountability and transparency. The World Health Organization's Declaration of Alma Ata, for example, states that "... people have the right and duty to participate individually and collectively in the planning and implementation of their health care" [7].

However, there is little evidence of the effects of engaging the public in health policy [4,5,8-12]. Of the 42 papers identified in a systematic review of public involvement in the planning and development of health care, for instance, 31 of these (74%) were case studies [8]. Often these papers attributed the involvement of the public to changes in services, including attempts to make services more accessible. Changes in the attitudes of organisations to involving the public and positive responses from consumers who took part in initiatives were also reported. Although this evidence suggested that public participation may have contributed to changes in the provision of services, such evidence was limited and came almost entirely from high-income countries.

**Figure 1:**
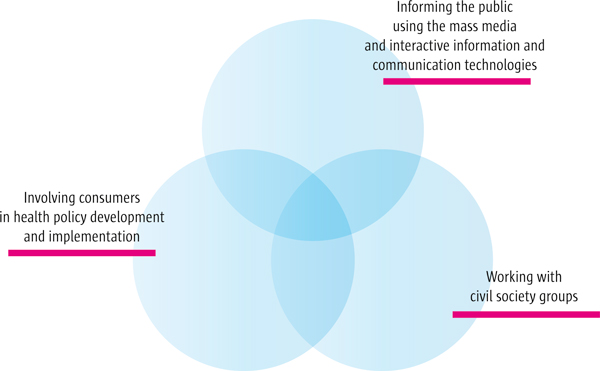
**Strategies to engage the public in evidence-informed health policymaking**.

When considering strategies to inform and engage the public in health policy development and implementation, it may be helpful to consider three broad strategy categories: those for working with the mass media (including the use of interactive information and communication technologies), those for working with civil society groups (organisations representing various interests), and those related to consumer involvement. As illustrated in Figure 1, effective engagement of the public in evidence-informed health policymaking is likely to require a combination of these overlapping strategies.

## Questions to consider

The following questions can be considered when developing and implementing health policies:

1. What strategies can be used when working with the mass media to inform the public regarding policy development and implementation?

2. What strategies can be used when working with civil society groups to inform and engage them in policy development and implementation?

3. What methods can be used to involve consumers in policy development and implementation?

### 1. What strategies can be used when working with the mass media to inform the public regarding policy development and implementation?

One way in which the public can be informed about (and potentially engaged in) evidence-informed health policy development is through the use of reports in the mass media. These reports are able to receive wide coverage and are an important source of information for the public, for healthcare professionals, and for policymakers. Although the impact of healthcare reporting is difficult to measure, mass media can influence individual health behaviours, levels of healthcare utilisation, healthcare practices, and health policy [13-16]. Nevertheless, health technology assessment (HTA) agencies, clinical practice guideline developers and units that support the use of research evidence in health policy have, thus far, generally made negligible efforts to communicate evidence to the wider public in this way [17].

Journalists are likely to agree that the accurate reporting of research related to health policy is important. However, many are faced with constraints that may limit their ability to achieve this goal [18,19]. These obstacles may include a lack of time, publishable space (or airtime) and knowledge; competition for audiences; difficulties with understanding and communicating jargon; problems with finding and using sources; problems with editors (who rarely have research training and may inhibit the ability to report research accurately); and commercial pressures (the need for journalists to sell their stories). As a consequence, much health reporting is either inaccurate or incomplete [20-24].

Mutual efforts by researchers and journalists are therefore likely to be needed to address these constraints, and may entail using a variety of strategies, including training, or innovations such as structured press releases [25]. Well-designed press releases could help to address the lack of time, space and knowledge within the mass media, as well as difficulties journalists may have with understanding jargon. It is, however, unclear whether such strategies could result in greater coverage for particular health issues [26]. Understanding the constraints which journalists face may also contribute to the design of more effective communication strategies. These could, for example, reflect recognition of the competing pressures of publishing space and audiences, as well as issues related to finding and using sources, or problems with editorial control. Efforts that do not recognise these constraints in the mass media are unlikely to be effective.

Possible strategies for working with the media to inform the public about the development and implementation of evidence-informed health policies include:

• *Structured press releases*: research press releases do not routinely highlight study limitations, and data are often presented using formats that may exaggerate the perceived importance of findings [22,26]. Presentations comparable to the format of the structured abstracts used in many journals (which include a section for the contextual description of the results, a section highlighting any limitations, and a statement about potential conflicts of interest) could help to ensure that journalists are given - and are therefore more likely to report - key information related to impact evaluations or other policy-relevant research. Press releases for policy-relevant systematic reviews could help to place research in context and shift the focus of reporting from the latest (but often misleading) single study to a broader understanding of newsworthy research relevant to important policy decisions [27,28]. Structures could also be used that are similar to those provided in the summaries of systematic reviews including, for example, key messages, a summary of key findings, and a description of the basis for the information used [29-32]. Similarly, press releases for policy briefs might use a structure that mirrors the contents of a policy brief, including structured sections with information about how a problem is defined, the relevant policy options and implementation strategies, and summaries of the key messages about the underlying evidence [33]

• *Fact boxes*: information is often not reported about the benefits and harms of clinical interventions and policy options, or it is reported in ways that are uninformative or may be misleading [22,23,26,34]. Standardised tables, similar to a summary of findings tables, that quantify the probability of outcomes together with different treatments or policy options [35,36], could also be used to enhance an understanding of the benefits, harms and costs of different options, and the extent to which we can be confident about those consequences [32,37,38]

• *Press conferences*: providing opportunities to question those involved in policy development and decisions may offer added value to journalists. The effectiveness of press conferences can be maximised by: planning ahead (two to three weeks where possible), timing the conference to achieve maximum coverage (e.g. holding it in the morning for a suitable length of time, ensuring that the conference does not clash with other events), issuing invitations that include all the relevant facts well in advance, ensuring easy access to the press conference, preparing a press kit (including a structured press release, fact boxes, relevant background material, and suitable illustrations), and ensuring that presentations are appropriately simple and have clear messages [39]

• *Providing stories*: it is important for journalists to be able to tell a story that will appeal to their audiences and also be both easily understood and informative. Providing journalists with appropriate anecdotes can facilitate this. These can play a complementary role in research and can facilitate the application of research evidence in health care decisions [40]. Anecdotes can also be vehicles enabling the delivery of research results to policymakers and health professionals, as well as to the public. It is, however, important to ensure that anecdotes are used appropriately to personalise and illustrate research findings and to present information in more meaningful ways. Conversely, it is important to ensure that anecdotes do not conflict with the available evidence

• *Avoiding jargon*: unnecessary jargon should be avoided in order to improve communication with journalists and the public in turn. In instances where terminology is necessary or useful, a glossary or the inclusion of fact boxes can help to explain essential terms and thereby help to improve reporting on important health policy issues. Another strategy is to write about issues in plain language first and then to introduce the relevant technical terms. This allows readers to understand technical concepts more clearly before seeing complex technical detail. The more common alternative of using the technical term first and providing a definition later, presents a barrier to immediate understanding and interrupts the flow of reading and assimilation of ideas

• *Providing access to experts*: to facilitate good coverage of important health policy issues it is important to identify people with relevant expertise, including researchers who are familiar with the research in question, as well as policymakers, stakeholders and people with a good understanding of relevant research or policy development methods. Briefing experts who are familiar with the media and can guide communication with the media may help to ensure that key information is delivered in ways that are understandable

• *Tip sheets*: providing journalists with simple questions to consider and discuss when they are interviewing experts, or researching or writing up stories, can help to ensure that key questions are asked about health policy issues and options, and that the answers are reported [21,41,42]

• *Training*: workshops or other types of training can help journalists gain greater understanding of evidence-informed health policymaking and to develop skills that may help to improve the quality of health policy reporting [19,43]. As a consequence, the extent to which the public is well-informed and better able to engage in the development and implementation of health policies may be improved

In addition to working with traditional mass media, consideration can be given to using new interactive information and communication technologies (ICTs) - including websites, blogs, and social networking sites - which are becoming more and more important. However, a lack of Internet access in some communities, particularly in low- and middle-income countries, limits access to online public engagement platforms. Infrastructural and cultural contexts vary and require different models and approaches. In addition, although the Internet is an important and increasingly popular source of information, policymakers face the challenge (similar to those in other forms of mass media) of competing with vast amounts of health information, some of which is neither accurate nor complete [35,44]. Harnessing the full potential of ICTs to engage the public in evidence-informed health policymaking therefore requires a mix of old and new technologies and thoughtful planning.

An OECD report on the challenges of online citizen engagement [45] proposes the following 10 strategies to guide online consultation:

1. Start planning early

2. Demonstrate commitment to the online consultation and communicate this clearly

3. Guarantee personal data protection

4. Tailor your approach to fit your target group

5. Integrate online consultation with traditional methods

6. Test and adapt tools (e.g. software, questionnaires)

7. Promote your online consultation

8. Ensure that sufficient time, resources and expertise are available to provide thorough analysis of the input received in the course of the online consultation

9. Publish the results of the online consultation as soon as possible and inform participants of the next steps in the policymaking process. Ensure that participants are informed of how the results were used in reaching decisions

10. Evaluate the consultation process and its impacts

### 2. What strategies can be used when working with civil society groups to inform and engage them in policy development and implementation?

Civil society can be defined in a number of ways. In this article, we use this term to refer to the wide range of organisations outside the state. These may include patient organisations, community groups, coalitions, advocacy groups, faith-based organisations, charities or voluntary organisations, professional associations, trade unions, and business associations.

**Figure 2:**
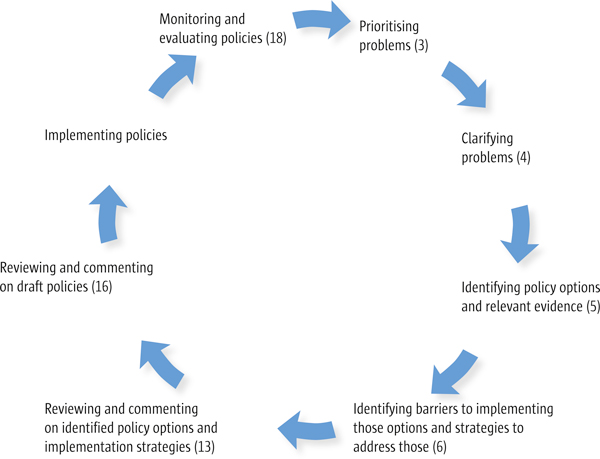
**Engagement of civil society in stages in the policy development and implementation cycle**. The numbers shown in brackets refer to the articles in this series (described in the Introduction [1]) which address the use of research evidence to inform each stage in the cycle

The National Institute for Health and Clinical Excellence (NICE), based in the United Kingdom, has adopted a comprehensive approach to involving the public and has a programme with dedicated staff responsible for public involvement [46-49]. NICE's efforts to involve stakeholder organisations are far more extensive than that of other clinical guideline developers. We are not aware of similar programmes aimed at engaging stakeholder organisations or civil society in the development or implementation of evidence-informed health policymaking. All of the strategies used by NICE, however, could potentially be applied to the engagement of civil society in evidence-informed health policymaking. Civil society, for example, could potentially be engaged in comparable stages for the development and implementation of health policies, as illustrated in Figure 2.

NICE includes the following organisations as stakeholders in its clinical guideline development process [50]:

• National patient and carer organisations that directly or indirectly represent the interests of people whose care is covered by each guideline ('patient and carer stakeholders')

• National organisations that represent healthcare professionals who provide the services described in each guideline ('professional stakeholders')

• Companies that manufacture the medicines or devices used in the clinical area covered by each guideline and whose interests may be significantly affected by each guideline ('commercial stakeholders')

• Providers and commissioners of health services in England, Wales and Northern Ireland

• Statutory organisations including the Department of Health, the Welsh Assembly Government, National Health Service (NHS) Quality Improvement Scotland, the Healthcare Commission, and the National Patient Safety Agency

• Research organisations that have done nationally-recognised research in each relevant area

It may be important to engage a broader list of civil society or stakeholder organisations for those health policies that focus on health systems arrangements including, for example, trade unions and business associations. NICE alerts potential stakeholder organisations in a number of ways and invites them to register their interest. These alerts include the issuing of press releases, listing topics on their website with details of how to register, contacting organisations that have registered for previous guidance to alert them to new topics, and writing to other patient, carer and professional organisations that may have an interest. NICE then contacts registered stakeholders and encourages them to get involved in the development of the different stages of such guidance. These include determining the scope of guidance, submitting evidence, commenting on draft guidance, and checking guidance revisions prior to publication.

Politicians and their constituency offices are likely to be familiar with the potential challenges of working with civil society, including claims that particular groups represent relevant patients or the public. Not all such groups do so adequately. Many patient groups are primarily advocacy groups that focus on obtaining resources for their particular area of interest or on providing peer support, rather than engaging in broader health policy issues [51]. And many patient organisations are funded by industry and may therefore also have conflicting interests [52]. Professional organisations, too, may have similar conflicts of interest. Some, for example, may receive funds from industry and be concerned primarily with the effects of policies on their own members rather than on health or the wider health care system.

### 3. What methods can be used to involve consumers in policy development and implementation?

Useful frameworks for describing and considering approaches to consumer involvement have been developed including, for example, the framework presented by Oliver and colleagues [4,5]. Similarly, Telford and colleagues have also developed a set of principles and indicators for involvement [53]. While both of these approaches have been developed in the context of consumer involvement in research, they provide useful frameworks for considering public engagement in health policy development and implementation.

The framework developed by Oliver and colleagues (Table 1) characterises diverse methods for involving consumers based on the degree of involvement, the forum for communication, involvement in decision making, the recruitment of representatives, training, and financial support.

**Table 1:** A framework for describing and considering approaches to consumer involvement*

Characteristics of different approaches	Examples
Degree of consumer involvement	• Consultation
	• Collaboration
	• Consumer control

Forum for communication	• Written consultation
	• Interviews
	• Focus groups
	• Consumer panels
	• Committee membership

Involvement in decision making	• No involvement
	• Implicit involvement
	• Explicit involvement

Recruitment	• Targeted, personal invitations
	• Wide advertising
	• Use of mass media
	• Contact by telephone, mail or email

Training and support	• Education (e.g. workshops)
	• Counselling
	• Mentoring
	• Introduction days

Financial support	• No financial support
	• Reimbursement of expenses
	• Fee or honoraria

* Adapted from Oliver and colleagues [4]

In their framework, the degree of involvement is classified in three ways, namely *consultation*, *collaboration *and *consumer control*. The process of *consultation *entails asking consumers for their views and using these to inform decision making. Policymakers or researchers, for example, may hold one-off meetings with consumers to ascertain their priorities or may write to consumers in accessible terms to invite their views. Consumers' views, in such instances, are not necessarily adopted although they may inform the decisions taken.

*Collaboration *entails active, ongoing partnerships with consumers. For example, consumers may be committee members (e.g. on the boards of health service organisations or regulatory committees) or they may collaborate less formally. Again, there is no guarantee that consumers' views will influence decisions, but collaboration offers more opportunities for them to be heard than consultation. Formal methods of decision making may help to ensure appropriate forms of collaboration [54]. Without these it may be difficult to judge whether public involvement has had any influence at all.

*Consumer control*, the third kind of consumer involvement in the framework, entails consumers developing and advocating or implementing health policies themselves. Professionals are only involved at the invitation of the consumers. In the context of public health systems this might entail, for example, the inclusion of politicians who are elected to represent their constituents.

Within this framework, methods are further distinguished by descriptions of the forum for communication (such as one-to-one interviews, focus groups, citizens' juries, town meetings, committee meetings, and working groups) and methods for decision making (such as informal committee consensus or voting). The presence or absence of transparent descriptions of methods for decision making can distinguish implied involvement in decisions (such as participation in committee meetings) and explicit involvement in decisions. Without transparent decision making there is a risk that consumer involvement may be regarded as tokenism.

Telford and colleagues used a consensus process to identify principles and indicators of successful consumer involvement [53]. Each of the eight principles they identified can be measured by at least one clear indicator (see Table 2). Although developed specifically to address the involvement of consumers in research, these principles and indicators are also relevant to public engagement in policy development and implementation.

**Table 2:** Principles and indicators of successful consumer involvement*

Principles	Indicators
The roles of consumers are agreed	• The roles of consumers were documented

The cost of consumer involvement is budgeted for	• Consumers were reimbursed for their travel
	• Consumers were reimbursed for their indirect costs (e.g. carer costs)

Policymakers respect the differing skills, knowledge and experience of consumers	• The contribution of consumers was reported

Consumers are offered training and personal support to enable their involvement	• Consumers were provided with training to enable their involvement

Policymakers ensure that they have the necessary skills to involve consumers effectively	• Policymakers were provided with training to enable them to involve consumers effectively

Consumers are involved in decision making	• Consumers' advice was documented
	• Consumers' role in decision making was documented

Consumer involvement is described in policy briefs	• Consumers' contributions were described and acknowledged in policy briefs
Policy briefs are available to consumers in formats and languages they can easily understand	• Summaries of policy briefs were disseminated to consumers in appropriate formats

* Adapted from Telford and colleagues [53]

## Conclusion

Policymakers, and those who support them, need to tailor strategies for engaging the public in evidence-informed policymaking to fit specific contexts, policies and key target groups. In poor countries, for example, radio may be the most important mass media. The Internet offers new opportunities for the interactive engagement of large numbers of consumers in policy development and decisions, and new ways to keep the public informed. Access to the Internet, however, varies widely. It is therefore important that the use of the Internet as a participation tool should be supplemented with other strategies in order to avoid exacerbating inequities in public engagement.

For the public to be effectively engaged in evidence-informed policymaking - and to avoid accusations of token involvement and consultation - it is important that policymakers and those who support them carefully plan and evaluate the strategies they use.

## Resources

### Useful documents and further reading

- Organisational self-assessment and planning tool for consumer and community participation: a tool for organisations involved in health policy and education. Version 1.0. 2003. http://www.healthissuescentre.org.au/documents/items/2008/05/208317-upload-00001.pdf

- Crawford MJ, Rutter D, Manley C, Weaver T, Bhui K, Fulop N, et al. Systematic review of involving patients in the planning and development of health care. BMJ 2002;325:1263-7

- Nilsen ES, Myrhaug HT, Johansen M, Oliver S, Oxman AD. Methods of consumer involvement in developing healthcare policy and research, clinical practice guidelines and patient information material. Cochrane Database of Systematic Reviews 2006, Issue 3

### Links to websites

- International Alliance of Patients' Organizations (IAPO): http://www.patientsorganizations.org - A global alliance of patients' organisations working at international, regional, national and local levels to represent and support patients, their families and carers.

- Association of Health Care Journalists (AHCJ): http://www.healthjournalism.org/index.php - An independent, non-profit organisation dedicated to advancing public understanding of health care issues. Its mission is to improve the quality, accuracy and visibility of health care reporting, writing and editing.

- INVOLVE is a national advisory group, funded by the National Institute for Health Research (NIHR): http://www.invo.org.uk/index.asp - Supports and promotes active public involvement in NHS, public health and social care research.

## Competing interests

The authors declare that they have no competing interests.

## Authors' contributions

ADO prepared the first draft of this article. SL, JNL and AF contributed to drafting and revising it.

## Supplementary Material

Additional file 1 Glossary Click here for file
